# Isolation, identification and antimicrobial sensitivity of *Ornithobacterium rhinotracheale* in broilers chicken flocks of Khuzestan, Iran

**Published:** 2016-12-15

**Authors:** Mansour Mayahi, Darioush Gharibi, Rahim Ghadimipour, Forough Talazadeh

**Affiliations:** 1 Department of Clinical Sciences, Faculty of Veterinary Medicine, Shahid Chamran University of Ahvaz, Ahvaz, Iran; 2 Department of Pathobiology, Faculty of Veterinary Medicine, Shahid Chamran University of Ahvaz, Ahvaz, Iran; 3 PhD Candidate of Bacteriology, Department of Pathobiology, Faculty of Veterinary Medicine, Shahid Chamran University of Ahvaz, Ahvaz, Iran; 4 Department of Research and Development, Razi Vaccine and Serum Research Institute, Agricultural Research Education and Extension Organization (AREEO), Ahvaz, Iran

**Keywords:** Antimicrobial sensitivity, Broiler chicken, Iran, *Ornithobacterium rhinotracheale*, Polymerase Chain Reaction

## Abstract

*Ornithobacterium rhinotracheale *(ORT) is a bacterium associated with respiratory disease, growth retardation, decreased egg production and mortality in chickens and turkeys. The objective of this study was isolation, identification and evaluation of antimicrobial susceptibility of ORT bacterium in slaughtered broilers chicken flocks based on cultural and molecular tests in Khuzestan province, south-west of Iran. A total of 210 tracheal swab samples were collected from 21 broiler flocks slaughtered in abattoirs of the province. The results of cultural and biochemical tests showed that 23 (10.95%) isolates from tracheal swabs of 4 flocks (19.04%) were identified as ORT, but according to molecular characterization, 18 (8.57%) ORT isolates were positive in PCR assay and produced the predicted 784 bp amplification product. Finally, using the disk diffusion method, the drug resistance patterns of ORT isolates were determined against a panel of commonly used antimicrobial agents. Antimicrobial susceptibility test revealed that all isolates (100%) were sensitive to tetracycline, florfenicol and cephalexin. The highest antimicrobial resistance (89.00%) was seen for fosfomycin, sultrim and gentamicin. The results of present research showed that there was significant difference between the isolation rates of ORT from various areas of the province. As well, our findings indicated that the simultaneous use of both cultural and molecular techniques results in more comprehensive outcomes in the isolation and identification of the organismfrom understudy hosts.

## Introduction

*Ornithobacterium rhinotracheale* (ORT) is a highly pleomorphic gram-negative and rod-shaped bacterium which causes important economic loss in poultry industry.^1^ It has been isolated from geese, fowls, chickens, ducks, turkeys, pigeons, partridges, chukar, partridges, ostriches, pheasants, quails, guinea gulls and rooks.^2^ Respiratory disease caused by ORT has been reported worldwide, although the severity of disease varies greatly depending on management factors and the presence of concurrent infections. The disease is characterized by airsacculitis, tracheitis and fibrinous pneumonia in severely affected birds. Mortality has been reported to be up to 10.00% in infected flocks and the high carcass condemnation rates lead to marked economic loss to producers. Other respiratory pathogens such as *E. coli *or *Bordetella avium *are often isolated with ORT and contribute to the clinical lesions observed.^1^^,^^3^

There are reports of ORT infections in many countries.^4^^-^^7^ In Iran, ORT infection was reported by Banani *et al. *for the first time. ^8^ Consequently, the results of serological studies from poultry flocks indicate that ORT is a relatively common pathogen in respiratory cases and occurs in different regions of the country.^9^^-^^12^ So far, a study using both cultural and molecular methods to identify the organism has not been carried out in the south-west areas of Iran, especially in Khuzestan province. The aim of the present research was isolation, identification and evaluation of antibiotic resistance profile of ORT by both biochemical and molecular methods in slaughtered broiler chicken flocks of Khuzestan province, Iran.

## Materials and Methods

**Sample collection. **A total of 210 tracheal swab samples were randomly collected from different 21 slaughtered broilers chicken flocks, with or without respiratory signs, in abattoirs of Khuzestan province, southwest of Iran, during the period of June to December 2015. Samples were transferred to Department of Pathobiology, Shahid Chamran University of Ahvaz, in test tubes with Cary-Blair transport medium (Becton-Dickinson, Maryland, USA) and in special sterile ice-filled containers to reserve the bacteria and prevent swabs from drying out after sampling.

**Bacteriological examinations. **Samples were streaked on 5% sheep blood agar with 10 μg mL^-1^ of gentamicin. Plates were incubated in a moist chamber with 7.5% CO_2_ at 37 ˚Cfor 24 to 48 hr.^1^ The pinpoint, circular, small, opaque to grayish and non-hemolytic colonies with 1 to 3 mm diameter were selected. Colonies with characteristics of ORT were stained by Gram staining and identified biochemically to confirm the main ORT characteristics and genetically by polymerase chain reaction (PCR). Biochemical characterization was assessed through oxidase, catalase, ability to growth on MacConkey agar (Merck, Darmstadt, Germany), H_2_S production in triple sugar iron (TSI) agar (Merck), indole production, urease, nitrate reduction, gelatinase and motility tests. Some carbohydrate fermentation tests such as sucrose, glucose, sorbitol, lactose, arabinose and maltose were also implemented.^1^^,^^13^^-^^15^ Suspected ORT isolates were stored in brain heart infusion (BHI, Merck) broth with 30% glycerol at –70 ˚C.

**Molecular characterization. **DNA extraction was performed on individual colonies which were suspended in 200 μL of sterile distilled water, heated at 100 ˚Cfor 10 min and then centrifuged for 10 min at 13000 rpm. 100 μL of supernatant fluid were used for molecular tests and frozen at –20 ˚Cuntil further uses.

**Polymerase chain reaction assay. **Primers used in this study were designed by Van Empel and Hafez.^2^ The sequences of primers were as follows: OR 16S-F1 (5´- GAG AAT TAA TTT ACG GAT TAA G-3´) and OR 16S-R1 (5´- TTC GCT TGG TCT CCG AAG AT-3´) which amplify a 784 bp fragment on the 16s rRNA gene of ORT. The PCR was performed in a mastercycler gradient thermocycler (Eppendorf, Hamburg, Germany) in a total reaction volume of 25 μL containing 5 μL of DNA template sample, 1.50 μL of each primer (10 pmol), 0.50 μL dNTP mix (10 mM), 1.50 μL MgCl_2_ (25 mM), 2.50 μL PCR buffer (10X) and 0.50 μL Taq DNA polymerase (1.25 units). All reagents were purchased from SinaClon Bioscience Co., Tehran, Iran. Amplification was obtained with an initial denaturation step at 94 ˚C for 7 min followed by 30 cycles at 94 ˚C for 30 sec (denaturation), 53 ˚C for 1 min (annealing) and 72 ˚C for 2 min. The final extension cycle was at 72 ˚C for 7 min. 10 μL of PCR products were separated by electrophoresis (100 volts for 1 hr) in a 1% agarose gel (CinnaGen Co., Tehran, Iran) stained with 0.50 μg mL^-1^ safe stain. DNA fragments were visualized by UV transillumination (UVitec, Cambridge, UK) and compared with a 100 bp DNA ladder. *Ornithobacterium rhinotracheale* serotype A and distilled water were used as positive and negative control, respectively. The positive control was obtained from the culture collection of the Department of Pathobiology, Shahid Chamran University of Ahvaz, Ahvaz, Iran.

**Antimicrobial susceptibility. **The test of the ORT isolates was determined by disk-diffusion method according to the procedures outline in the Clinical and laboratory standards institute document M31-A3.^16^ Briefly, bacterial isolates were taken from 24 hr blood agar culture plates. The inoculums were prepared by making a direct suspension of isolated colonies from agar plates in tryptic soy broth (TSB; Merck) and then applied with a sterile cotton swabs on surface of Mueller-Hinton agar (Merck) with 5% sheep blood. During this test, the sensitivity of the isolates was evaluated over the discs of 15 different antimicrobial agents (Padtan Teb Co., Tehran, Iran). After 20 hr of incubation at 37 ˚C, the measurement of inhibition (halo) zones and the interpretation of results were made according to the guidelines described in CLSI: M31-A3.

**Statistical analysis. **The results were analyzed statistically by using SPSS software (version 19.0; SPSS Inc., Chicago, USA). Descriptive statistics were computed to determine the proportions of the isolation of bacteria in different areas and ratio of isolates resistant to different antimicrobial agents. Chi-square test was pursued for determination of statistical significance of differences between the proportions and *p*-values of < 0.05 were considered statistically significant.

## Results

**Fig. 1.**
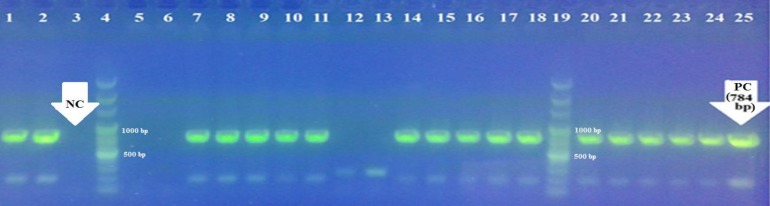
Electrophoresis of PCR products on 1% agarose gel stained with safe stain. **Lanes 4 and 19:** 100 bp molecular weight marker; **Lane 25:** Positive control; **Lane 3:** Negative control; **Lanes 5-6 and 12-13: **Negative samples; **Lanes 1-2, 7-11, 14-18 and 20-24:** *Ornithobacterium rhinotracheale* specific 784 bp band

 The results of cultural and biochemical tests showed that 23 isolates from tracheal swabs of four flocks (19.04% out of 21 broiler flocks and 10.95% out of 210 tracheal swabs) were identified as ORT, but according to molecular characterization, 18 (8.57%) ORT isolates were positive in PCR assay and produced the predicted 784 bp amplification product (Table 1 and Fig. 1). After 24 hr of incubation on blood agar, pinpoint grey to grey/ white colonies were observed, becoming considerably larger after 48 hr of incubation. The colonies were non-hemolytic on blood agar plates. The unique characteristic of the colonies was their poor adherence to agar. They showed no growth on MacConkey and TSI agars and were non-motile. The gram stain revealed presence of gram-negative, pleomorphic and rod-shaped micro-organisms and according to biochemical tests, isolated organisms were negative for catalase, indole, urease and gelatinase, but were positive for oxidase. They fermented sucrose, glucose, lactose, arabinose and maltose but not sorbitol (Table 2).

**Table 1:** Results of detection and identification of *Ornithobacterium rhinotracheale *from totally 210 tracheal swab samples from 21 slaughtered broilers chicken flocks (10 sample from each flock), by cultural tests and polymerase chain reaction technique, in Khuzestan province during the period of June to December 2015

**Area (city)**	**Flocks size**	**Age (days)**	**Healthy**	**Affected**	**Positive** ** on culture**	**Positive ** **on ** **PCR **
**Shooshtar**	20000	42	■	□	0	0
**Dezfool**	25000	48	■	□	0	0
	10000	45	■	□	1	1
	20000	42	□	■	2	2
	36000	45	■	□	1	0
	20000	42	■	□	10	10
	20000	50	■	□	6	5
**Andimeshk**	40000	42	■	□	0	0
	36000	45	■	□	0	0
**Shadeghan**	20000	48	■	□	0	0
	10000	42	■	□	0	0
**Masjedsoleyman**	10000	49	■	□	0	0
**Laaly**	20000	44	□	■	1	0
	10000	45	■	□	2	0
**Baghmalek**	20000	45	□	■	0	0
**Behbahan**	20000	42	■	□	0	0
	30000	48	■	□	0	0
**Hendyjan**	30000	46	■	□	0	0
**Ramshyr**	20000	50	□	■	0	0
**Dashtazadeghan**	40000	45	■	□	0	0
**Maahshahr**	10000	46	■	□	0	0
**Total**	467000	-	-	-	23(10.95%)	18(8.57%)

**Table 2:** Results of cultural and biochemical tests used to identify the *Ornithobacterium rhinotracheale* isolated from broilers chicken flocks in Khuzestan province during the period of June to December 2015

**Test**	**Result**
**Growth on ** **blood agar** *	+
**Hemolysis**	-
**Growth on MacConkey agar**	-
**H**_2_ **S production** **triple sugar iron agar**	-
**Gram staining**	-
**Catalase**	-
**Oxidase**	+
**Indole production**	-
**Urease**	-
**Nitrate** ** reduction**	-
**Gelatinase**	-
**Motility**	-
**Acid from carbohydrates:**	
**Glucose**	+
**Lactose**	+
**Maltose**	+
**Sucrose**	+
**Arabinose**	+
**Sorbitol**	-

* with 10 μg mL^-1^ gentamicin.

The results of antimicrobial susceptibility test of the isolates are shown in Table 3. All the isolates (100%) were susceptible to tetracycline, florfenicol and cephalexin and 89.00% of the isolates were resistant to fosfomycin, sultrim and gentamicin, but 11 (61.10%) and 6 (33.30%) isolates were moderately sensitive to nalidixic acid and enrofloxacin, respectively (Table 3). All of the positive samples (100%) were from broiler flocks of Dezfool city (south-west Iran), (Table 1). These findings showed that there was significant difference between the rates of ORT isolation from various areas of Khuzestan province (*p* < 0.05).

**Table 3:** Antimicrobial susceptibility test results of 18 *Ornitho-bacterium rhinotracheale *strains isolated from slaughtered broilers chicken flocks in Khuzestan province during the period of June to December 2015

**Antimicrobial agent**	**Number of isolates**		
	**Susceptible**	**Intermediate**	**Resistant**
**Gentamicin (** **10 µg** **)**	1 (5.50%)	1 (5.50%)	16 (89.00%)
**Enrofloxacin (** **5 µg** ** )**	12 (66.60%)	6 (33.30%)	0 (0.00%)
**Flumequin (** **30 µg** **)**	14 (77.70%)	2 (11.10%)	2 (11.10%)
**Tylosin (** **30 µg** **)**	16 (89.00%)	0 (0.00%)	2 (11.10%)
**Tetracycline (** **30 µg** **)**	18 (100%)	0 (0.00%)	0 (0.00%)
**Lincospectin (109 ** **µg** **)**	14 (77.70%)	2 (11.10%)	2 (11.10%)
**Penicillin (** **10 µg** **)**	15 (83.30%)	1 (5.50%)	2 (11.10%)
**Florfenicol (** **30 µg** **)**	18 (100%)	0 (0.00%)	0 (0.00%)
**Neomycin (** **30 µg** **)**	6 (33.30%)	0 (0.00%)	12 (66.60%)
**Streptomycin (** **10 µg** **)**	8 (44.40%)	2 (11.10%)	8 (44.40%)
**Nalidixic acid (** **30 µg** **)**	3(16.60%)	11 (61.10%)	4 (22.20%)
**Fosfomycin (** **200 µg** **)**	2 (11.10%)	0 (0.00%)	16 (89.00%)
**Sultrim (25 ** **µg** **)**	2(11.10%)	0 (0.00%)	16 (89.00%)
**Cephalexin (** **30 µg** **)**	18 (100%)	0 (0.00%)	0 (0.00%)
**Cloxacilline (** **1 µg** **)**	4 (22.20%)	3 (16.60%)	11(61.10%)

## Discussion

 Respiratory signs in ORT infection are not pathognomonic and clinical signs of disease are also associated with other respiratory diseases. Diagnosis of ORT infection should be based on the isolation and identification of the agent. Isolation of ORT from various species of domesticated and wild birds has been the subject of studies in many countries. The frequencies of the organism in other studies were different from our results. Ozbey *et al.* examined 250 lung and trachea samples taken from 10 slaughtered broiler chicken flocks with respiratory manifestations in Turkey and reported isolation of ORT from tracheas of 5 (1.50%) chickens and from both lung and trachea of only 1 (0.40%) chicken in the cultural and PCR assays.^17^ Also, reportedly ORT was isolated less-frequently (0.40% and 1.20%, respectively) from tracheal swab and tracheal tissue samples collected from broiler chickens in other areas of Turkey.^18^^,^^19^ Whereas, Roussan *et al.* reported that 21 out of 100 (21.00%) tracheal swabs collected from commercial broiler flocks with respiratory disease in southern and northern areas of Jordan were positive for ORT in PCR test.^20^

Hassanzadeh *et al.* reported that following biochemical and PCR assays, ORT was isolated and characterized from 1 out of 150 (0.60%) tracheal swab samples and 3 out of 300 (1.00%) total lung and tracheal tissue samples collected from broiler chickens in slaughterhouses and dead birds of broiler flocks with respiratory diseases symptoms, respectively, from north, west and center of Iran.^14^ Similarly, Asadpour *et al.* reported that 3 out of 290 (1.03%) tracheal swab samples from 29 slaughtered broiler chicken flocks in Guilan province (north of Iran) were positive for ORT by cultural and PCR methods.^21^ As well, Barin *et al.* examined tracheal swab samples collected from 38 broiler chicken flocks affected with respiratory disorders in Babol city (north of Iran) by cultural method and reported that only 1 (2.60%) flock was positive for ORT.^22^ Also, Seifi reported that from 450 tracheal samples collected from 45 broiler flocks in Mazandaran province (north of Iran), 12 (2.60%) ORT isolates were identified using biochemical tests.^23^

In comparison with above studies, the results of present research indicated that the rate of ORT presence was high in broiler chicken flocks of Khuzestan province. In agreement with our findings, Ghaemmaghami *et al.* examined 173 trachea and lung samples taken from broiler chicken flocks with clinical respiratory signs in Markazi province (center of Iran) by biochemical tests and reported that ORT was isolated from 17 (9.80%) samples.^24^ Similarly, Jamshidian and Mayahi reported that 22 out of 254 (8.66%) tracheal swab samples from slaughtered broiler chicken flocks in Khuzestan province were positive for ORT by biochemical and cultural tests.^25^ However, Banani *et al.* examined tracheal samples from carcasses of 100 broilers, broiler breeders and layer flocks with respiratory disorders and showed that 59 (59.00%) isolates were identified as ORT, which were higher than our results.^26^ Canal *et al*. collected 1550 sera related to 50 slaughtered broiler flocks in southern Brazil and showed the high prevalence (63.83%) of ORT antibodies.^27^ Also, Chansiripornchai *et al*. randomly examined 17 broiler farms (19 flocks) in Thailand.^28^ 63.00% of flocks were seropositive and the sera analysis on individual 280 broiler sera showed that the antibody responses were 19.60% positive. In the West Azerbaijan (north-west of Iran), Allymehr examined 463 sera from 50 broiler flocks.^11^ The result showed that 41 broiler flocks (82.00%) were positive for ORT. Similarly, Ganbarpour and Salehi reported the sero-prevalence of ORT in the south east of Iran.^29^ 134 (31.90%) out of 420 serum samples or 17 (81.00%) out of 21 broiler flocks were positive. The chance of ORT isolation is more at early stages of the infection and its recovery at later stages often fails. In contaminated samples, ORT can be hidden easily by overgrowth of other bacteria and therefore, cannot be detected in routine investigations.^1^^,^^30^

 The multi-drug resistance emergence in ORT strain is one of the major veterinary concerns. The antibiotic resistance profile of the different strains of ORT depends on the more commonly used antibiotics in the source of their isolation. In Germany and the Netherlands, in contrast to our results, most ORT isolates are resistant to enrofloxacin.^4^ Similarly, in Canada, pure ORT has been isolated from enrofloxacin-treated birds.^5^ Whereas, in agreement with our findings, Devriese *et al*. reported that 98.00% of ORT strains isolated in Belgium were sensitive to quinolones specially enrofloxacin, *in vitro.*^31^ Also, Soriano *et al.* reported that susceptibility of Mexican isolates of ORT to amoxicillin, enrofloxacin and oxytetracycline was variable.^32^ It has been shown that depending on the ORT strain’s isolation source, strains have very variable susceptibility to antimicrobials.^1^^,^^33^ In Iran, in contrast to our results, Ghaemmaghami *et al.* found that most of the isolated ORT organisms were resistant to tylosin and lincospectin and the isolates were sensitive to florfenicol, enrofloxacin, trimethoprim-sulfamethoxazole, ceftiofur, chloramphenicol and flumequin, respectively.^24^ Similarly, according to Asadpour *et al.*, all of ORT isolates (100%) were resistant to enrofloxacin, ciprofloxacin, erythromycin, tetracycline, flumequin, lincospectin and furazolidon and all of them were susceptible to tiamulin and ceftriaxon, but two isolates (66.70%) were moderately sensitive to tylosin and amoxicillin and sensitive to florfenicol.^21^ Barin *et al.* reported the tested ORT isolates were resistant to nalidixic acid, sultrim, streptomycin, gentamycin, tetracycline, colistin, furazolidon and flumequin. ^22^ Also, Jamshidian and Mayahi showed that all of ORT isolates were resistant to gentamycin and trimethoprim–sulfamethoxazole and all of them were found to be susceptible to ampicillin and chloramphenicol.^25^ As well as, Mirzaie *et al.* found that danofloxacin and chloramphenicol show antimicrobial activities against all ORT strains.^34^ Like the ORT strains isolated in other studies, the strains examined in this research have been shown to be resistant to some of the major antibiotics, perhaps due to inappropriate use of antibiotics for treatment of secondary infections related to the prevalence of respiratory diseases complex in broiler chicken farms.

On the basis of our results, the rate of ORT presence was high in broiler chicken flocks in studied district. Also, our findings showed that there was significant difference between the isolation rates of ORT from various areas of the province. As well, all the isolated organisms were susceptible to tetracycline, florfenicol and cephalexin. Commonly, the sensitivity pattern of ORT strains depends on the source of the strain and routinely used antibiotics in the area.^2^ In general, the simultaneous use of both cultural and molecular techniques results in more comprehensive outcomes in isolation and identification of the organismfrom understudy hosts.

## References

[1] Markey BK, Leonard FC, Archambault M et al (2013). Clinical veterinary microbiology.

[2] Van Empel PC, Hafez MH (1999). Ornithobacterium rhinotracheale. Avian Pathol.

[3] Hoerr FJ (2010). Clinical aspects of immunosuppression in poultry. Avian Dis.

[4] Van Beek P (1994). Ornithobacterium rhinotracheale (ORT), clinical aspects in broilers and turkeys. Annual meeting of the veterinary study group of the EU, Amsterdam.

[5] Joubert P, Higgins R, Laperle A (1999). Isolation of Ornithobacterium rhinotracheale from turkeys in Quebec, Canada. Avian Dis.

[6] Sakai E, Tokuyma Y, Nonaka F (2000). Ornithobacterium rhinotracheale infection in Japan: Preliminary investigations. Vet Record.

[7] Turan N, Ak S (2002). Investigation of the presence of Ornithobacterium rhinotracheale in chickens in Turkey and determination of the seroprevalence of the infection using the enzyme-linked immunosorbent assay. Avian Dis.

[8] Banani M, Khaki P, Goodarzi H (2000). Isolation and identification of Ornithobacterium rhinotracheale from a broiler and a pullet flock [Persian]. Pajouhesh-va-Sazandegi.

[9] Banani M, Pourbakhsh SA, Khaki P (2003). Serotyping and drug sensitivity of Salmonellae isolates from commercial chickens and domestic pigeons submitted to Razi institute [Persian]. Pajouhesh-va-Sazandegi.

[10] Banani M, Pourbakhsh SA, Khaki P (2004). Isolation and identification of bacterial agents in commercial chickens suffered from swollen head and face. Iran J Vet Res.

[11] Allymehr M (2006). Seroprevalence of Ornithobacterium rhinotracheale infection in broiler and broiler breeder chickens in West Azerbaijan province, Iran. J Vet Med.

[12] Asadpour Y, Bozorgmehrifard MH, Pourbakhsh SA (2008). Isolation and identification of Ornithobacterium rhinotracheale in broiler breeder flocks of Guilan province, north of Iran. Pakistan J Biol Sci.

[13] Banani M, Pourbakhsh SA, Erami M (2009). Diagnosis of Ornithobacterium rhinotracheale using polymerase chain reaction (PCR). J Vet Res.

[14] Hassanzadeh M, Karimi V, Fallah N (2010). Molecular characterization of Ornithobacterium rhinotracheale isolated from broiler chicken flocks of Iran. Turk J Vet Anim Sci.

[15] Van Empel PCM, Van Den Bosch H, Loeffen P (1997). Identification and serotyping of Ornithobacterium rhinotracheale. J Clinic Microbiol.

[16] Clinical and laboratory standards institute (CLSI) (2008). Performance standards for antimicrobial disk and dilution susceptibility test for bacteria isolated from animals; Approved Standard, 3rd ed. CLSI document M31-A3.

[17] Ozbey G, Ongor H, Balik DT (2004). Investigations on Ornithobacterium rhinotracheale in broiler flocks in Elazig province located in the East of Turkey. Vet Med-Czech.

[18] Erganis O, Hadimli HH, Kav K (2002). A comparative study on detection of Ornithobacterium rhinotracheale antibodies in meat type turkeys by dot immuno binding assay, rapid agglutination test and serum agglutination test. Avian Pathol.

[19] Turkyilmaz S (2005). Isolation and serotyping of Ornitho-bacterium rhinotracheale from poultry. Turk J Vet Anim Sci.

[20] Roussan DA, Al-Rifai RH, Khawaldeh GY (2011). Ornithobacterium rhinotracheale and Mycoplasma synoviae in broiler chickens in Jordan. Rev Sci Tech.

[21] Asadpour Y, Banani M, Pourbakhsh SA (2011). Isolation, identification and antibiotic sensitivity determination of Ornithobacterium rhinotracheale in slaughtering broiler chicken flocks of Guilan province. Iran J Vet Res.

[22] Barin M, Pourbakhsh SA, Banani M (2008). Isolation, identification and in vitro antibiotic sensitivity of Ornithobacterium rhinotracheale from Babol commercial broiler flocks [Persian]. Vet J Islamic Azad Univ Garmsar Branch.

[23] Seifi S (2012). Seroprevalece and isolation of Ornithobacterium rhinotrachealein broiler flocks in Mazandaran province, north of Iran. Bulgar J Vet Med.

[24] Ghaemmaghami SSH, VandeYousefi J, Niroumand H (2007). Survey of prevalence of Ornithobacterium rhinotracheale in broiler farms affected with respiratory disorders in Markazi province [Persian]. J Vet Res.

[25] Jamshidian M, Mayahi M (2008). Isolation of Ornithobacterium rhinotracheale from broilers in Ahvaz [Persian]. Iran Vet J.

[26] Banani M, Pourbakhsh SA, Khaki P (2001). Characterization of Ornithobacterium rhinotracheale isolates from commercial chickens. Arc Razi Inst.

[27] Canal CW, Leao JA, Ferreira DJ (2003). Prevalence of antibodies against Ornithobacterium rhinotracheale in broilers and breeders in southern Brazil. Avian Dis.

[28] Chansiripornchai N, Wanasawaeng W, Sasipreeyajan J (2007). Seroprevalence and identification of Ornithobacterium rhinotracheale from broiler and broiler breeder flocks in Thailand. Avian Dis.

[29] Ghanbarpour R, Salehi M (2009). Seroprevalence and identification of Ornithobacterium rhinotracheale in broiler flocks in south-eastern Iran. Tropic Anim Health Prod.

[30] Hafez H (2002). Diagnosis of Ornithobacterium rhinotracheale. Int J Poult Sci.

[31] Devriese LA, Hommez J, Vandamme P (1995). In vitro antibiotic sensitivity of Ornithobacterium rhino-tracheale strains from poultry and wild birds. Vet Record.

[32] Soriano V, Vera N, Salado C (2003). In vitro susceptibility of Ornithobacterium rhinotracheale to several anti-microbial drugs. Avian Dis.

[33] Tsai HJ, Huang CW (2006). Phenotypic and molecular characterization of isolates of Ornithobacterium rhinotracheale from chickens and pigeons in Taiwan. Avian Dis.

[34] Mirzaie S, Hassanzdeh M, Bozorgmehrifard MH (2011). Isolation and characterization of Ornithobacterium rhinotracheale in the commercial turkey, quail flocks and domestic pigeons by bacteriological and molecular methods. Arc Razi Inst.

